# Intravenous dexmedetomidine versus tramadol for treatment of shivering after spinal anesthesia: a meta-analysis of randomized controlled trials

**DOI:** 10.1186/s12871-020-01020-y

**Published:** 2020-05-04

**Authors:** Jinguo Wang, Zaitang Wang, Junyan Liu, Na Wang

**Affiliations:** 1 Department of Urology, The First Hospital of Jilin University, No.1 Xinmin Street, Changchun, Jilin, 130021 China; 2 Department of Taxation, School of Public Economics and Administration of Shanghai University of Finance and Economics, NO.777, Guoding Road, Yangpu District, Shanghai, 200433 China; 3 Department of Anesthesiology, The First Hospital of Jilin University, No.1 Xinmin Street, Changchun, Jilin, 130021 China

**Keywords:** Dexmedetomidine, Tramadol, Postanesthesia shivering, Meta-analysis

## Abstract

**Background:**

Shivering is a frequent complication after spinal anesthesia. Increasing studies have compared the effect of intravenous dexmedetomidine and intravenous tramadol on shivering after spinal anesthesia, hence we performed a meta-analysis of randomized controlled trials to compare dexmedetomidine with tramadol on the treatment of post-spinal anesthesia shivering.

**Methods:**

PubMed, Embase, Cochrane library, Web of Science and Google Scholar were searched to find the eligible studies comparing the effect of dexmedetomidine and tramadol on the treatment of shivering after spinal anesthesia. Mean difference (MD) or risk ratio (RR) along with 95% confidence interval (CI) was used to analyze the outcomes. I^2^ test was conducted to assess the heterogeneity of the included trials. We utilized Review Manager 5.3 to perform statistical analyses.

**Results:**

Thirteen randomized controlled trials including 864 subjects were included. Dexmedetomidine had higher effective rate of shivering control (RR =1.03; 95%CI [1.01, 1.06], *P* = 0.01, I^2^ = 14%), shorter time to cease shivering (MD = -2.14; 95%CI [− 2.79, − 1.49], *P* < 0.00001, I^2^ = 98%), lower recurrent rate of shivering (RR = 0.45; 95%CI [0.27, 0.73], *P* = 0.001, I^2^ = 0%), lower incidences of nausea (RR = 0.10; 95%CI [0.05, 0.19], P < 0.00001, I^2^ = 48%), and vomiting (RR = 0.13; 95%CI [0.06, 0.30], P < 0.00001, I^2^ = 0%), higher incidence of sedation (RR = 2.48; 95%CI [1.32, 4.65], *P* = 0.005, I^2^ = 82%), hypotension (RR = 2.50; 95%CI [1.24, 5.03], *P* = 0.01, I^2^ = 0%) and bradycardia (RR = 4.78; 95%CI [1.76, 13.00], *P* = 0.002, I^2^ = 0%), compared with tramadol.

**Conclusions:**

Dexmedetomidine is superior to tramadol for shivering treatment, due to higher effective rate of shivering control, earlier onset of action and lesser recurrence of shivering with higher incidence of sedation and lower incidences of nausea and vomiting. However, dexmedetomidine is also associated with higher incidences of hypotension and bradycardia than tramadol.

## Background

Shivering is a common perioperative complication because of postanesthesia hypothermia [1]. Spinal anesthesia has impairment of shivering in the block area and greater heat loss than general anesthesia because of abnormal heat loss owing to vasodilatation [2, 3]. Shivering can cause severe consequences, such as arterial hypoxia and myocardial ischemia by increasing oxygen consumption [4, 5]. Tramadol is commonly used for the treatment of shivering in clinical practice. However, tramadol can lead to nausea and vomiting which is very distressing for the patient. Therefore, it is necessary to find a better drug with fewer side effects. Dexmedetomidine, an alpha 2-adrenergic agonist, has been confirmed the effect on treatment and prevention of shivering in various surgeries by reducing the shivering threshold [6].

There are no large-sample clinical trials evaluating the advantages or disadvantages between dexmedetomidine and tramadol on post-spinal anesthesia shivering. Therefore, we conduct a meta-analysis of randomized controlled trials (RCTs) to compare the effect of intravenous dexmedetomidine and tramadol on post-spinal anesthesia shivering.

## Methods

### Literature review

Relevant articles were found by searching PubMed, Cochrane library, Web of Science and Google Scholar by two investigators independently. The terms used for searching included: “Dex”, “Dexmedetomidine”, “Tramadol”, “Anesthesia, Spinal”, “Injections, Spinal” and “Shivering” through March 2020, without limits. Furthermore, the researchers looked through the references of the relative papers to find additional studies.

### Inclusion criteria of studies

Inclusion criteria were as follows: 1) the patients underwent an operation under spinal anesthesia or combined spinal and epidural anesthesia; 2) the comparison was between intravenous dexmedetomidine and tramadol about the treatment effect of shivering; 3) the incidence of side effects was reported in both dexmedetomidine and tramadol groups; 4) the study was a RCT. Meeting papers, correspondences and editorials were excluded.

### Data extraction

Data were collected independently by two researchers, including patient characteristics, types of surgery, anesthetic type, the drugs for spinal anesthesia, doses of the study drugs, shivering degree, efficacy of shivering treatment, incidence of recurrent shivering and adverse effects. Shivering was graded using a four point scale as per Wrench in all included papers [7]. Any disaccord was further settled by the third researcher.

### Evaluation of risk of bias and the study quality

Two researchers independently evaluated the risk of bias and the qualities of all included studies according to Cochrane Handbook v5.0.2 and 5 point Jadad scale [8]. Each of the following items of risk of bias was graded as “high risk of bias”, “uncertain risk of bias” or “low risk of bias”: random sequence generation, allocation concealment, blinding of participants and personnel, blinding of outcome assessment, incomplete outcome data, selective reporting and other bias. Disputes were settled by discussion, if necessary, a third investigator helped to make a decision.

### Statistical analysis

Review Manager 5.3 (Cochrane Collaboration, Copenhagen, Denmark) was utilized to perform all statistical analyses. For dichotomous data, risk ratio (RR) with 95% confidence interval (CI) was calculated with the Mantel-Haenszel method. Mean difference was used for continuous variables. If there was significant heterogeneity (I^2^ > 50%), we tried to find possible reasons of heterogeneity, and then sensitivity analysis was performed with fixed effect model.

## Results

**Fig. 1:**
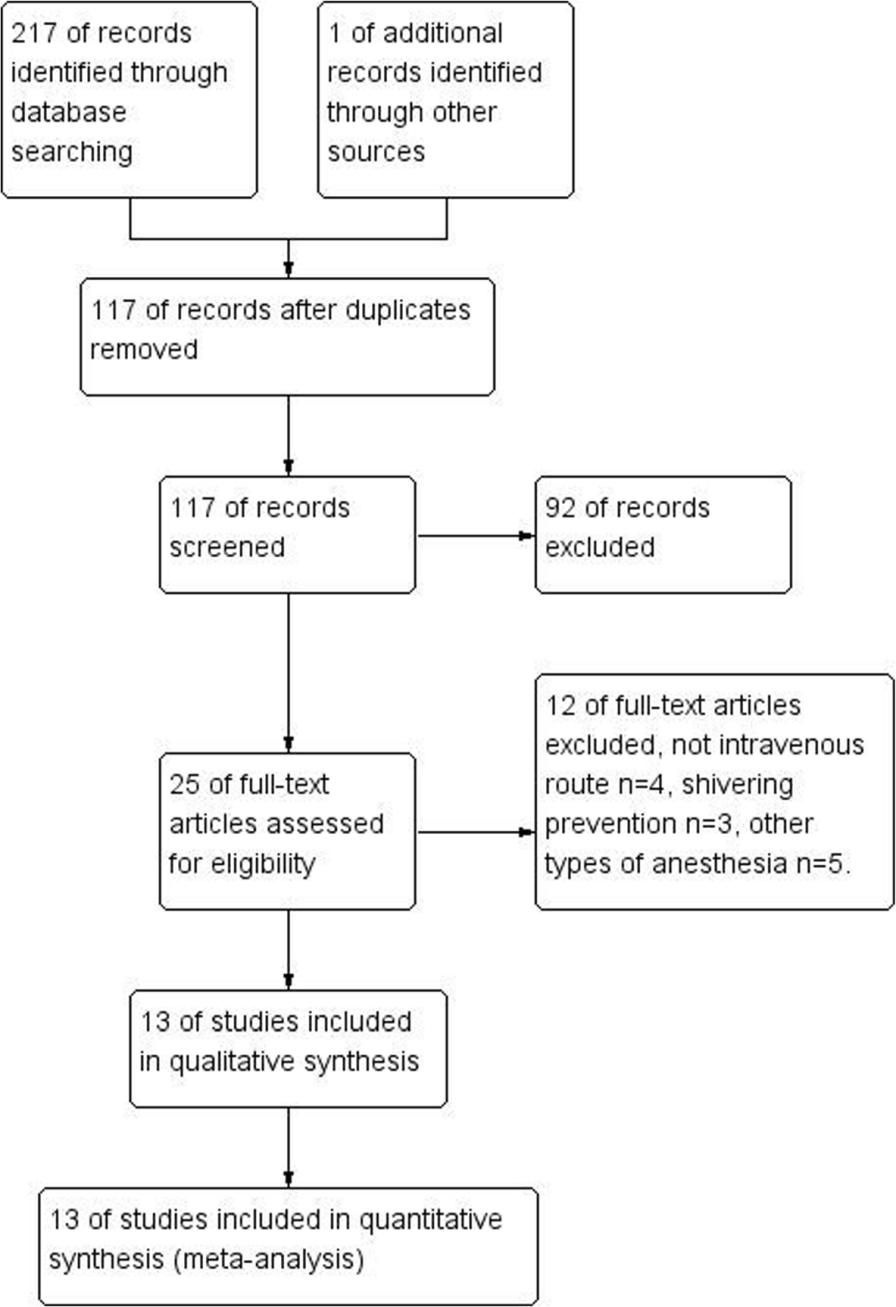
The flow chart of study selection

**Fig. 2:**
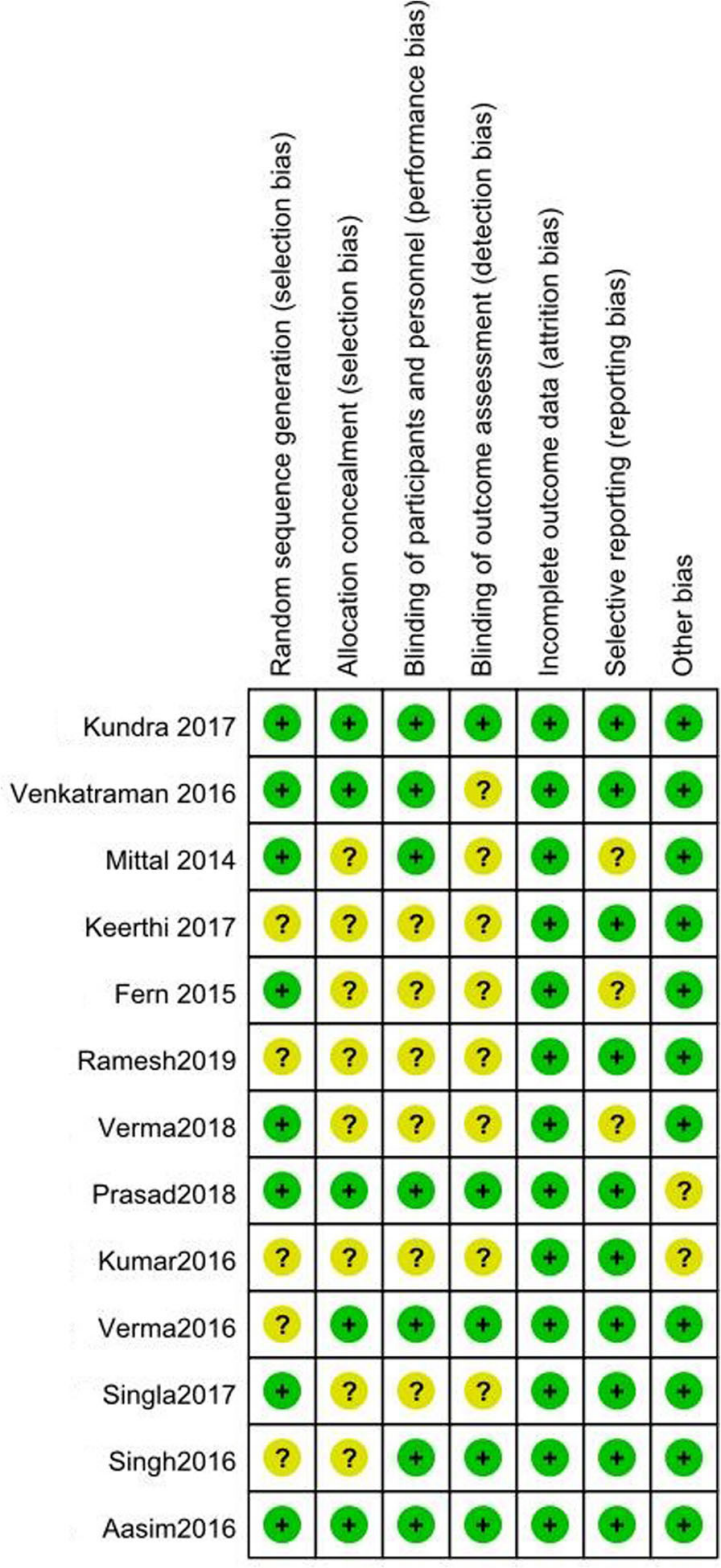
The risk of bias assessment of the included studies. Note: There was no high risk of bias found in these studies

Figure 1 showed the flow chart of this meta-analysis. Thirteen studies were included, involving 864 patients (432 received dexmedetomidine and 432 tramadol) [9–21]. The characteristics of the identified clinical trials were displayed in Tables 1. Surgeries were performed under spinal anesthesia in 12 studies [9–20] and under combined spinal and epidural anesthesia in one study [21]. In the included studies, 7 compared dexmedetomidine with tramadol [9–15] and the other 6 compared dexmedetomidine with tramadol and clonidine [16–18, 20], pethidine [21] or butorphanol [19]. Because our study only compared dexmedetomidine with tramadol, clonidine, pethidine and butorphanol were neglected. The risk-of-bias plot was formed utilizing Review Manager 5.3. (Fig. 2).

**Table 1:** Characteristics of the included studies

Author Date	Dosage	Study type	Jadad Score	Sample size D/T	Patient characteristics; Surgical setting	Type of anesthesia; Drug for anesthesia	Definition of shivering	Outcomesmeasures
Kundra 2017	Dexmedetomidine 0.5 μg/kg, tramadol 0.5 mg/kg	RCT	5	50/50	18–65 yr, ASA: I-II; Orthopedic, general, or urological surgery	Spinal anesthesia; 0.5% bupivacaine	Grade 3 or 4	①②③④⑤⑥⑦⑧
Venkatraman 2016	Dexmedetomidine 0.5 μg/kg, tramadol 1 mg/kg	RCT	5	30/30	18–70 yr, ASA: I-II; No mention	Spinal anesthesia; To achieve sensory level of at least T10	Grades 2 to 4	①②③⑤⑥⑦⑧
Mittal 2014	Dexmedetomidine 0.5 μg/kg; tramadol 0.5 mg/kg	RCT	4	25/25	18–65 yr, ASA: I-II; Lower abdominal, lower limb, orthopaedic and plastic surgeries	Spinal anesthesia; 0.5% heavy bupivacaine 15 mg.	Grade 3 or 4	①②③④⑤⑥⑦⑧
Fern 2015	Dexmedetomidine 0.5 μg/kg; tramadol 0.5 mg/kg	RCT	3	20/20	18–70 yr, ASA: I-II; Elective orthopaedic, gynaecology or general surgery	Combined spinal and epidural anesthesia; 0.5% hyperbaric bupivacaine 15 mg.	Grade 3 or 4	①②⑥⑦⑧
Keerthi 2017	Dexmedetomidine 0.5 μg/kg; tramadol 0.5 mg/kg	RCT	4	32 /32	18–60 yr, ASA: I-II; Lower abdomen and lower limb surgery	Spinal anesthesia; 0.5% hyperbaric bupivacaine 2.8 to 3 ml	Grades 2 to 4	①②③④⑤⑥⑦⑧
Ramesh 2019	Dexmedetomidine 0.5 μg/kg; tramadol 0.5 mg/kg	RCT	3	30/30	18–65 yr, ASA: I-II; Elective lower limb surgery	Spinal anesthesia; 0.5% hyperbaric bupivacaine 12.5 mg	Grade 3 for at least 2 min	①②③④⑤⑧
Verma 2018	Dexmedetomidine 0.5 μg/kg; tramadol 0.5 mg/kg	RCT	4	60/60	18–65 yr, ASA: I-II; Elective lower limb, lower abdominal, gynaecological procedures, caesarean sections	Spinal anesthesia; No mention	Grade 3 or 4	①②③④⑤⑥⑦⑧
Prasad 2018	Dexmedetomidine 0.5 μg/kg; tramadol 0.5 mg/kg	RCT	5	25/25	18–40 yr, ASA: I-II; Caesarean section	Spinal anesthesia; 0.5% hyperbaric bupivacaine	Grade 3 or 4	①②③④⑤⑥⑦⑧
Kumar 2016	Dexmedetomidine 0.6 μg/kg; tramadol 1.0 mg/kg	RCT	3	20/20	18–60 yr, ASA: I-II; Elective lower abdominal surgeries and lower limb surgeries	Spinal anesthesia; 0.5% hyperbaric bupivacaine 15 mg	Grade 3	①②③⑥⑦
Verma 2016	Dexmedetomidine 0.5 μg/kg; tramadol 2 mg/kg	RCT	4	30/30	18–45 yr, ASA: I-II; Elective abdominal, gynecological and orthopedic surgeries	Spinal anesthesia; 0.5% hyperbaric bupivacaine 15 mg	Grades 2 to 4	①②③④⑤⑥⑦
Singla 2017	Dexmedetomidine 0.5 μg/kg; tramadol 0.5 mg/kg	RCT	4	50/50	18–65 yr, ASA: I-II; Various surgeries under spinal anesthesia	Spinal anesthesia; 0.5% hyperbaric bupivacaine 3–3.6 ml	Grades 2 to 4	①②③⑥⑦⑧
Singh 2016	Dexmedetomidine 0.5 μg/kg; tramadol 0.5 mg/kg	RCT	4	30/30	20–50 yr, ASA: I-II; Elective lower abdominal, lower limb orthopaedic and gynaecological surgeries	Spinal anesthesia; 0.5% hyperbaric bupivacaine 15 mg	Grade 3	①②③④⑤⑥⑦⑧
Aasim 2016	Dexmedetomidine 0.5 μg/kg; tramadol 0.5 mg/kg	RCT	5	30/30	18–65 yr, ASA: I-II; Various surgeries under spinal anesthesia	Spinal anesthesia; 0.5% hyperbaric bupivacaine 15 mg	Grade 3	①②③④⑤⑥⑦⑧

*RCT* randomized controlled trial, *ASA* American Society of Anesthesiologists. ①: Effective rate of shivering treatment, ②: Time to cease shivering, ③: recurrent rate of shivering, ④: the incidence of nausea, ⑤: the incidence of vomiting, ⑥: the incidence of bradycardia, ⑦: the incidence of hypotension, ⑧: sedation score

### Effective rate

**Fig. 3:**
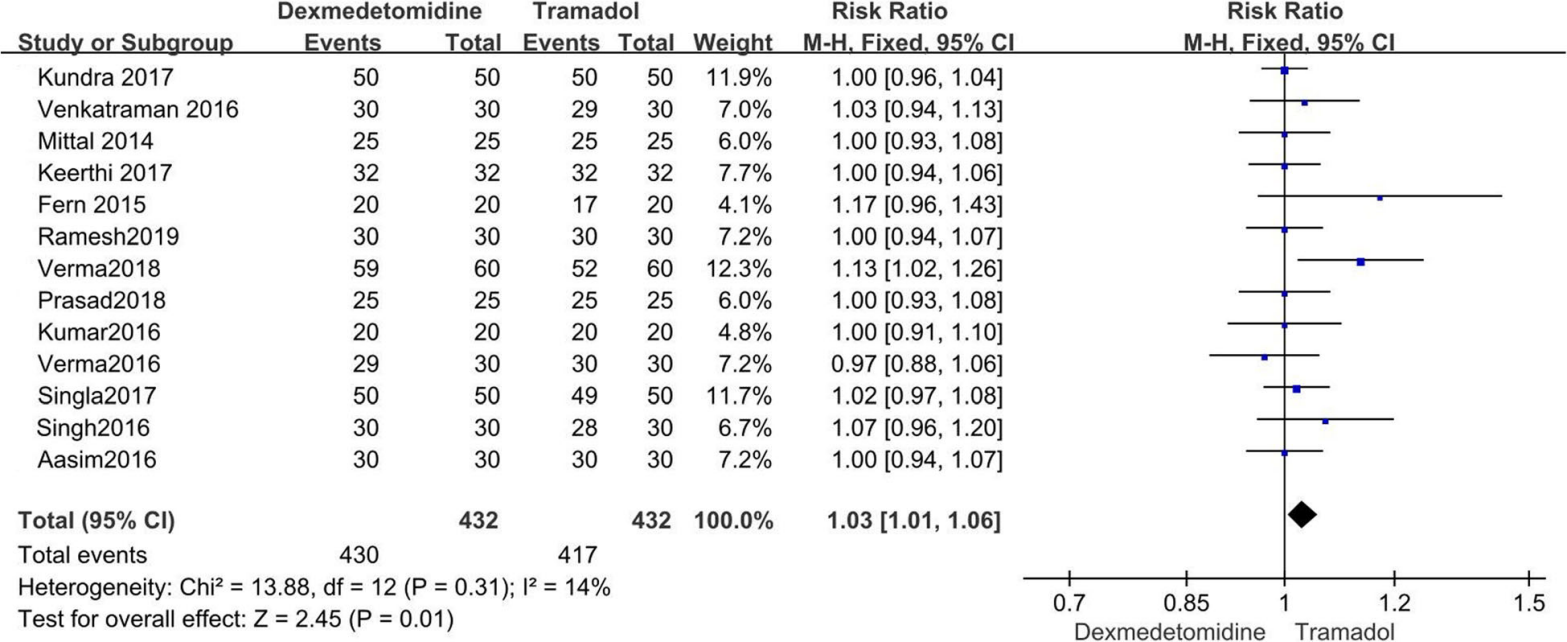
Forest plot for effective rate of shivering. Abbreviations: CI, confidence interval; M-H, Mantel-Haenszel

All eligible RCTs reported the effective rate of shivering control [9–21]. The value of I^2^ = 0% indicated no heterogeneity among the included studies. Dexmedetomidine had higher effective rate of shivering control than tramadol (RR =1.03; 95% CI [1.01, 1.06], *P* = 0.01, I^2^ = 14%). (Fig. 3).

### Time to cease shivering

**Fig. 4:**
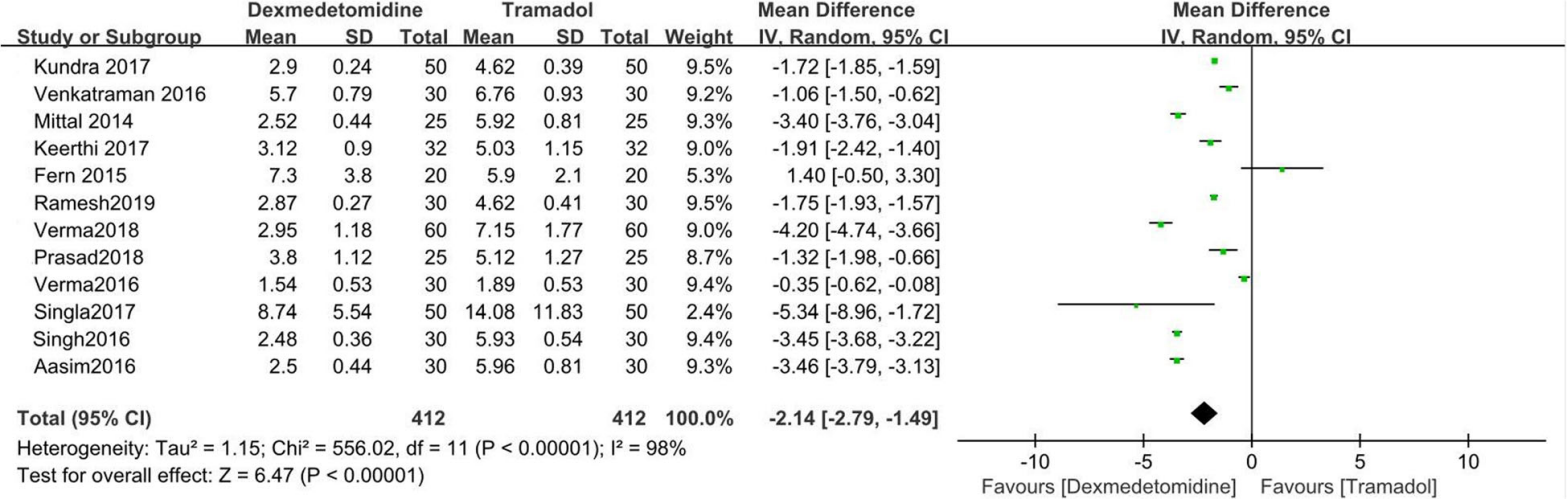
Forest plot for time to cease shivering in minutes. Abbreviations: SD, standard deviation; CI, confidence interval; IV, inverse variance

Twelve included RCTs compared time to cease shivering of dexmedetomidine and tramadol [9–16, 18–21]. The random effect model was utilized, because a high heterogeneity was detected (I^2^ = 98%). The result showed that dexmedetomidine was associated with shorter time to cease shivering than tramadol (MD = -2.14; 95%CI [− 2.79, − 1.49], *P* < 0.00001, I^2^ = 98%). (Fig. 4) Sensitivity analysis was performed for time to cease shivering by excluding single study sequentially, but no source of heterogeneity was detected.

### Recurrent rate of shivering

**Fig. 5:**
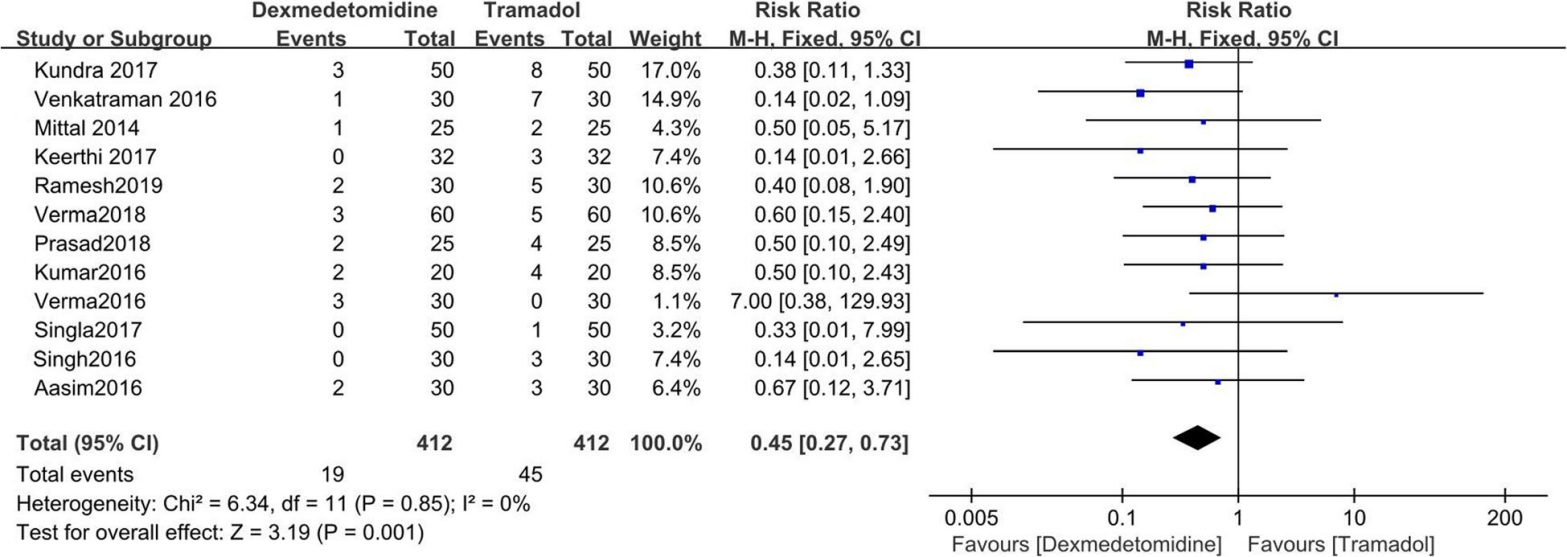
Forest plot comparing recurrent rate of shivering. Abbreviations: CI, confidence interval; M-H, Mantel-Haenszel

There were 12 studies reporting the recurrent rate of shivering [9–20]. The value of I^2^ = 0% indicated no heterogeneity. The result of this study indicated that the recurrent rate of shivering of tramadol was significantly higher than that of dexmedetomidine (RR = 0.45; 95%CI [0.27, 0.73], *P* = 0.001, I^2^ = 0%). (Fig. 5).

### Nausea and vomiting

**Fig. 6:**
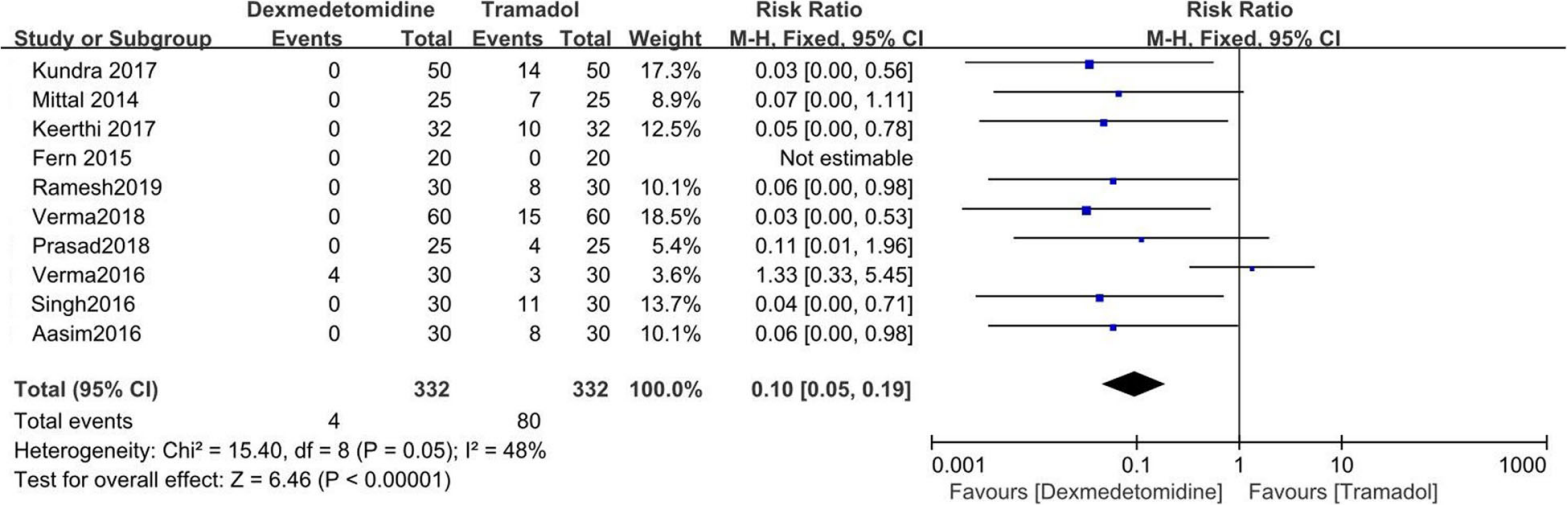
Forest plot comparing the incidence of nausea. Abbreviations: CI, confidence interval; M-H, Mantel-Haenszel

**Fig. 7:**
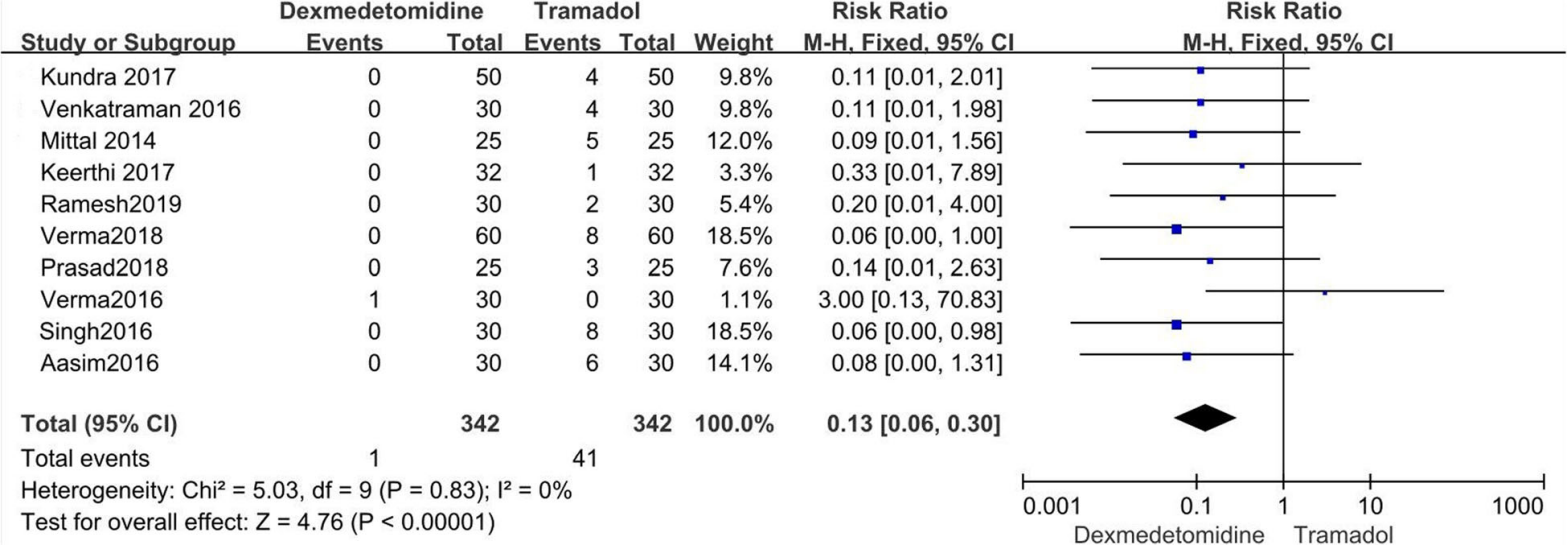
Forest plot comparing the incidence of vomiting. Abbreviations: CI, confidence interval; M-H, Mantel-Haenszel

Ten papers recorded nausea, [9–13, 15, 18–21] and 10 recorded vomiting [9–13, 15, 16, 18–20]. Four out of 332 patients receiving dexmedetomidine experienced nausea, and 80 out of 332 patients receiving tramadol experienced nausea. There were 342 patients receiving dexmedetomidine (1 with vomiting) and 342 patients receiving tramadol (41 with vomiting). Dexmedetomidine had lower incidences of nausea and vomiting than tramadol (Nausea: RR = 0.10; 95%CI [0.05, 0.19], *P* < 0.00001, I^2^ = 48%; Vomiting: RR = 0.13; 95% CI [0.06, 0.30], P < 0.00001, I^2^ = 0%). (Figs. 6 and 7).

### Hypotension and bradycardia

The incidences of hypotension and bradycardia were recorded in all of the included RCTs, but one [13]. There were 402 patients receiving dexmedetomidine (24 experienced hypotension and 19 had bradycardia) and 402 patients receiving tramadol (9 experienced hypotension and 2 had bradycardia).

**Fig. 8:**
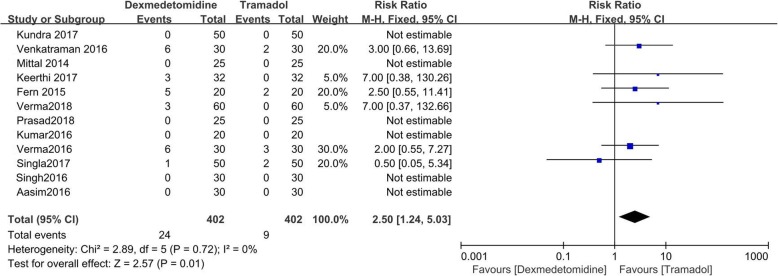
Forest plot comparing the incidence of hypotension. Abbreviations: CI, confidence interval; M-H, Mantel-Haenszel

**Fig. 9:**
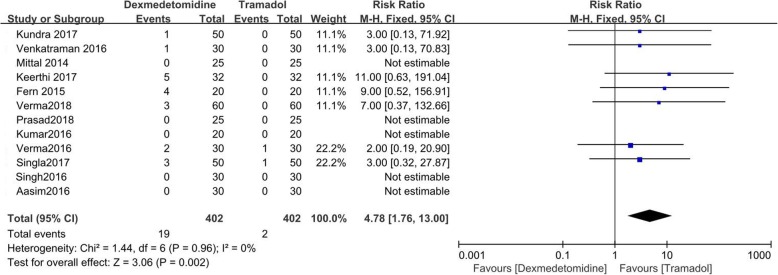
Forest plot comparing the incidence of bradycardia. Abbreviations: CI, confidence interval; M-H, Mantel-Haenszel

Dexmedetomidine was associated with higher incidence of hypotension (RR = 2.50; 95%CI [1.24, 5.03], *P* = 0.01, I^2^ = 0%), and bradycardia (RR = 4.78; 95%CI [1.76, 13.00], *P* = 0.002, I^2^ = 0%). (Figs. 8 and 9).

### Sedation

**Fig. 10:**
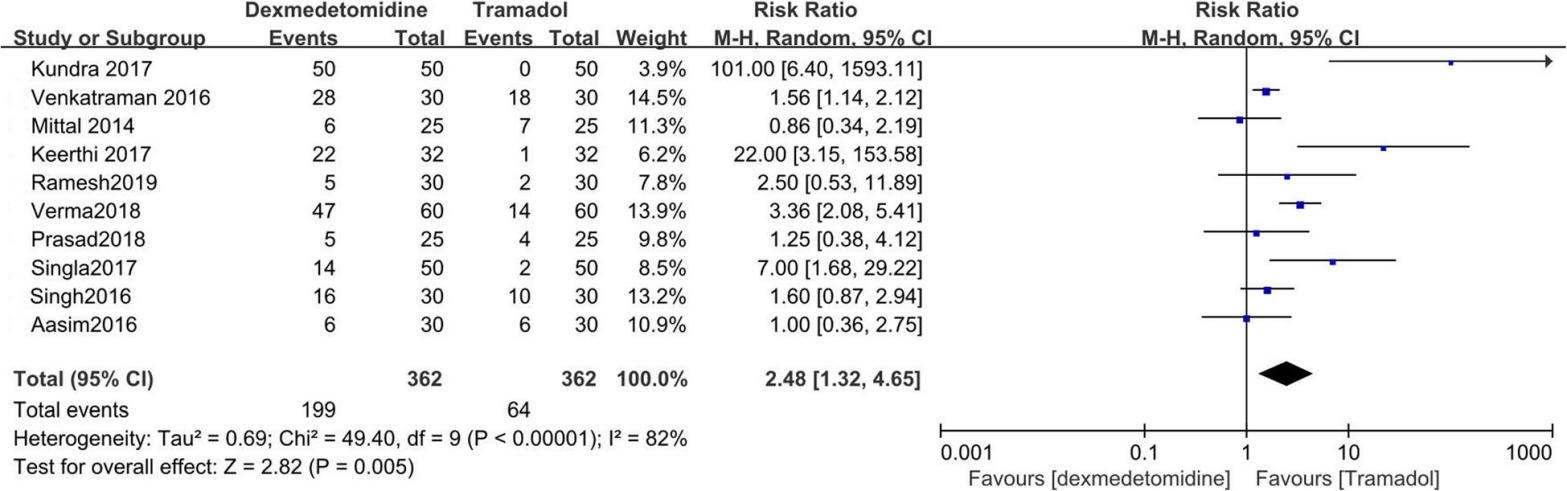
Forest plot comparing the incidence of sedation. Abbreviations: CI, confidence interval; M-H, Mantel-Haenszel

Ten studies reported the incidence of sedation which we defined as being drowsy and responding to verbal or physical stimuli [9–16, 19, 20]. The value of I^2^ = 82% indicated high heterogeneity. The incidence of sedation of dexmedetomidine was significantly higher than that of tramadol (RR = 2.48; 95%CI [1.32, 4.65], *P* = 0.005, I^2^ = 82%). (Fig. 10).

Sensitivity analysis was performed for the incidence of sedation by excluding single study sequentially, but no source of high heterogeneity was detected. There were no patients with over sedation reported in the included studies. Over sedation was defined as no response to physical stimuli.

### Publication bias

**Fig. 11:**
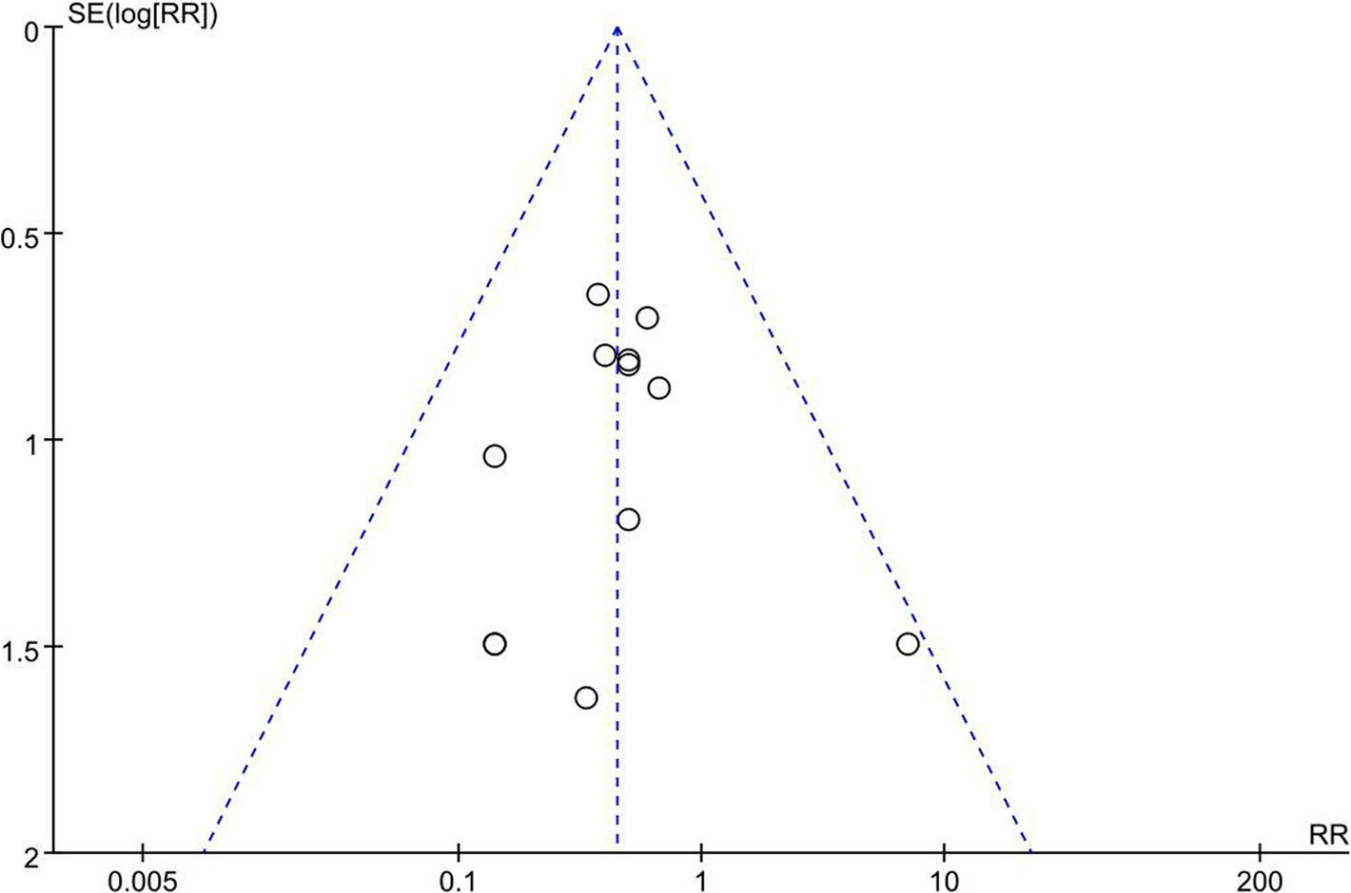
Funnel plot of recurrent rate of shivering

Figure 11 showed that no publication bias was detected for recurrent rate of shivering.

## Discussion

In this meta-analysis, we compare the efficacy of intravenous dexmedetomidine and tramadol on the treatment of shivering after spinal anesthesia in adult patients. Dexmedetomidine is associated with higher effective rate of shivering control, shorter time to cease shivering, lesser recurrence of shivering, lower incidences of nausea and vomiting, higher incidences of hypotension, bradycardia and sedation than tramadol.

In this meta-analysis, dexmedetomidine has shorter time to cease shivering and a higher incidence of sedation than tramadol. But these outcomes have high heterogeneities, which are possibly associated with the following: 1) Inclusion criteria for shivering degree are different among the included studies, shivering degrees of 2 to 4 are included in 4 RCTs [14, 16, 18, 19] and shivering degrees of 3 or 4 in the other 9 RCTs [9–13, 15, 17, 20, 21]. 2) The types and doses of local anesthetics for spinal anesthesia are different among the studies; 3) The types and duration of the surgeries are different.

In this study, tramadol is associated with significantly higher incidences of nausea and vomiting than dexmedetomidine. Nausea and vomiting is very distressing for the patient. Moreover, vomiting may cause rare but serious consequences, such as aspiration, esophageal rupture, subcutaneous emphysema or pneumothorax [22]. However, dexmedetomidine has significantly higher incidences of hypotension and bradycardia compared to tramadol. Dexmedetomidine has an inherent property of postsynaptic activation of alpha 2-adrenoceptors in the central nervous system to decrease heart rate and blood pressure. We can’t assess the clinically significance of hypotension and bradycardia, because of lack of research data.

Tramadol is a well-established agent in treatment of shivering. The mechanism of anti-shivering action of tramadol may be its opioid or serotonergic and noradrenergic activity or both [23–25]. Dexmedetomidine, an alpha-2 adrenoceptor agonist, has antihypertensive, sedative, analgesic and anti-shivering properties [26]. The anti-shivering effects of alpha adrenoceptor agonists are mediated by binding to alpha receptors that mediate the vasoconstriction. In addition, it has hypothalamic thermoregulatory effects of reducing the vasoconstriction and shivering thresholds [27]. It suggests that dexmedetomidine acts on the central thermoregulatory system rather than preventing shivering peripherally [28]. The effect of dexmedetomidine on treatment of shivering has been confirmed in the previous studies [29–31]. Dexmedetomidine and tramadol are not only effective for shivering treatment, but also effective for shivering prevention [32–34].

The sedation achieved is better in patients receiving dexmedetomidine than patients receiving tramadol. None of patients experiencing over sedation or respiratory depression is reported in the included studies [9–21]. Since the surgery is done under spinal anesthesia, the sedation seen with dexmedetomidine is beneficial for the surgeon, anesthetist as well as the patient, because it provides comfort and amnesia to the patient, cardiorespiratory stability and good surgical conditions during surgery. Therefore, dexmedetomidine may be a good choice for shivering control after spinal anesthesia because of its dual effects of anti-shivering and sedation.

There are two limitations in this study. First of all, there is a high heterogeneity regarding time to cease shivering and the incidence of sedation. Secondly, intravenous dexmedetomidine causes hypotension and bradycardia, but we haven’t analyzed whether it is clinically significant, due to lack of research data. Therefore, more RCTs are required for further study.

## Conclusions

Dexmedetomidine is superior to tramadol for shivering treatment, due to higher effective rate of shivering control, earlier onset of action and lesser recurrence of shivering with higher incidence of sedation and lower incidences of nausea and vomiting. However, dexmedetomidine is also associated with higher incidences of hypotension and bradycardia than tramadol.

## Data Availability

The datasets used and/or analysed during the current study are available from the corresponding author on reasonable request. All data generated or analyzed during this study can be found in PubMed, Embase, Cochrane library, Web of Science and Google Scholar.

## Abbreviations

MD: Mean difference
RR: Relative risk
CI: Confidence interval
RCT: Randomized controlled trials

## References

[1] Shukla U, Malhotra K, Prabhakar T (2011). A comparative study of the effect of clonidine and tramadol on post-spinal anaesthesia shivering. Indian J Anaesth..

[2] Alfonsi P (2003). Postanaesthetic shivering. Epidemiology, pathophysiology and approaches to prevention and management. Minerva Anestesiol.

[3] Crowley LJ, Buggy DJ (2008). Shivering and neuraxial anesthesia. Reg Anesth Pain Med.

[4] Giesbrecht GG, Sessler DI, Mekjavić IB, Schroeder M, Bristow GK (1994). Treatment of mild immersion hypothermia by direct body-to-body contact. J Appl Physiol.

[5] Ciofolo MJ, Clergue F, Devilliers C, Ben Ammar M, Viars P (1989). Changes in ventilation, oxygen uptake, and carbon dioxide output during recovery from isoflurane anesthesia. Anesthesiology..

[6] Blaine Easley R, Brady KM, Tobias JD (2007). Dexmedetomidine for the treatment of postanesthesia shivering in children. Pediatric Anaesth.

[7] Wrench IJ, Singh P, Dennis AR, Mahajan RP, Crossley AW (1997). The minimum effective doses of pethidine and doxapram in the treatment of post-anaesthetic shivering. Anaesthesia..

[8] Jadad AR, Moore RA, Carroll D, Jenkinson C, Reynolds DJ, Gavaghan DJ (1996). Assessing the quality of reports of randomized clinical trials: is blinding necessary?. Control Clin Trials.

[9] Kundra TS, Kuthiala G, Shrivastava A, Kaur P (2017). A comparative study on the efficacy of dexmedetomidine and tramadol on post-spinal anesthesia shivering. Saudi J Anaesth.

[10] Mittal G, Gupta K, Katyal S, Kaushal S (2014). Randomised double-blind comparative study of dexmedetomidine and tramadol for post-spinal anaesthesia shivering. Indian J Anaesth.

[11] Verma A, Bhandari D, Dhande P, Jain S, Tidke S (2018). Comparative evaluation of dexmedetomidine and tramadol for attenuation of post-Spinal anaesthesia shivering. J Clin Diagn Res.

[12] Prasad P (2019). Comparison of tramadol and dexmedetomidine for treatment of shivering during spinal anaesthesia in caesarean section. J Evolution Med Dent Sci.

[13] Ramesh K, Bhushanam KN, Sharma BA, Kumar SSS (2019). A clinical comparative study between intra venous dexmedetomidine and tramadol for control of post spinal anesthesia shivering. J Dent Med Sci.

[14] Singla A, Chaudhari M, Patel A (2017). Efficacy and safety of tramadol and dexmedetomidine in treatment of shivering following spinal anaesthesia: a randomized controlled study. J Med Sci Clin Res.

[15] Aasim SA, Reddy AS, Reddy V, reddy M, Chandrakanth C (2016). Randomised double-blind comparative study of tramadol and dexmedetomidine for post-spinal anaesthesia shivering. J Chalmeda Anand Rao Institut Med Sci.

[16] Venkatraman R, Karthik K, Pushparani A, Mahalakshmi A (2016). A prospective, randomized, double-blinded control study on comparison of tramadol, clonidine and dexmedetomidine for post spinal anesthesia shivering. Rev Bras Anestesiol.

[17] Kumar YD, Kumar TR, Sasi RK, Prachi P (2016). A clinical comparative study of dexmedetomidine, clonidine and tramadol in post spinal shivering in lower limb and lower abdominal surgeries. J Dent Med Sci.

[18] Verma NK, Kumar M (2016). Comparison of clonidine, dexmedetomidine and tramadol for control of post spinal shivering: A randomized double blind clinical study. Int J Life Sci Scienti Res.

[19] Keerthi P, Kamath SS (2017). Comparative study of dexmedetomidine, butorphanol and tramadol for post-spinal anesthesia shivering. Res J Pharmaceutical Biological Chemical Sci.

[20] Singh A, Gupta KK, Solanki SL, Kumar M (2016). A prospective randomized double blind comparative study of clonidine, dexmedtomidine and tramadol for treatment of shivering under spinal anaesthesia. Ind J Public Health Res Dev.

[21] Fern L, Misiran K (2015). Comparison of dexmedetomidine, pethidine and tramadol in the treatment of post-neuraxial anaesthesia shivering. South Afr J Anaesth Analg.

[22] Apfel CC (2010). Postoperative nausea and vomiting. Miller's Anaesthesia.

[23] Lee CR, McTavish D, Sorkin EM (1993). Tramadol. A preliminary review of its pharmacodynamic and pharmacokinetic properties, and therapeutic potential in acute and chronic pain states. Drugs..

[24] Mathews S, Al Mulla A, Varghese PK, Radim K, Mumtaz S (2002). Postanaesthetic shivering-a new look at tramadol. Anaesthesia..

[25] Tsai YC, Chu KS (2001). A comparison of tramadol, amitriptyline, and meperidine for postepidural anesthetic shivering in parturients. Anesth Analg.

[26] Grewal A (2011). Dexmedetomidine: new avenues. J Anaesthesiol Clin Pharmacol.

[27] Bajwa SJ, Bajwa SK, Kaur J, Singh G, Arora V, Gupta S (2011). Dexmedetomidine and clonidine in epidural anaesthesia: a comparative evaluation. Indian J Anaesth..

[28] Talke P, Tayefeh F, Sessler DI, Jeffrey R, Noursalehi M, Richardson C (1997). Dexmedetomidine does not alter the sweating threshold, but comparably and linearly decreases the vasoconstriction and shivering thresholds. Anesthesiology..

[29] Bajwa SJ, Gupta S, Kaur J, Singh A, Parmar S (2012). Reduction in the incidence of shivering with perioperative dexmedetomidine: a randomized prospective study. J Anaesthesiol Clin Pharmacol.

[30] Usta B, Gozdemir M, Demircioglu RI, Muslu B, Sert H, Yaldiz A (2011). Dexmedetomidine for the prevention of shivering during spinal anesthesia. Clinics (Sao Paulo).

[31] Moawad HES, Elawdy MM (2015). Efficacy of intrathecal dexmedetomidine in prevention of shivering in patients undergoing transurethral prostatectomy: a randomized controlled trial. Egypt J Anaesth.

[32] Ameta N, Jacob M, Hasnain S, Ramesh G (2018). Comparison of prophylactic use of ketamine, tramadol, and dexmedetomidine for prevention of shivering after spinal anesthesia. J Anaesthesiol Clin Pharmacol.

[33] Bozgeyik S, Mizrak A, Kılıç E, Yendi F, Ugur BK (2014). The effects of preemptive tramadol and dexmedetomidine on shivering during arthroscopy. Saudi J Anaesth.

[34] Singh S, Verma VK, Prasad C, Prakash J (2016). Randomised double-blind comparative study of dexmedetomidine and tramadol for prevention of perioperative shivering in transurethral resection of prostate under spinal anaesthesia. J Evolution Med Dent.

